# Mechanism of action of HMGB1 in urologic malignancies

**DOI:** 10.3389/fonc.2025.1593157

**Published:** 2025-09-03

**Authors:** Dandan Li, Xu Lei, Lanlan Zhao, Qingqing Fu, Yao Chen, Xiaorong Yang

**Affiliations:** 1 Department of Pathology, Affiliated Hospital of Zunyi Medical University, Zunyi, Guizhou, China; 2 School of Basic Medical Sciences, Zunyi Medical University, Zunyi, Guizhou, China; 3 Department of Urology, Third Affiliated Hospital of Zunyi Medical University, Zunyi, China

**Keywords:** high mobility group protein 1 (HMGB1), prostate cancer, renal cell carcinoma, urologic tumors, bladder cancer

## Abstract

High mobility group protein 1 (HMGB1) is a highly conserved chromatin-associated protein that is widely found in eukaryotic cells. Studies have shown that HMGB1 plays an important role in the development and progression of urological malignancies. As a classical damage-associated molecular pattern (DAMP), HMGB1 usually acts as a DNA chaperone in the nucleus. In response to external stimuli, HMGB1 is actively secreted by immune cells and can also be passively released into the extracellular space from necrotic cells. By interacting with various signaling pathways and receptors, HMGB1 can induce immune system activation and participate in carcinogenesis, metastasis and angiogenesis. This review systematically summarizes the role of recent research progress of HMGB1 in carcinogenesis, progression, prognosis and potential clinical applications of different urological malignancies, providing reference for the diagnosis and treatment of urological malignancies.

## 1 Introduction

High mobility group proteins (HMGs) are a class of highly conserved non-histone chromatin proteins first described by Graham and Clive in 1973 (1).The HMG family consists of HMGA, HMGN, and HMGB, of which HMGB1 (also known as HMG1 or amphotericin) is the most abundantly expressed protein in the HMG family and is widely expressed in mammalian cells. HMGB1 can change its intracellular location depending on the cell type, organization and external environment and performs a variety of roles. In the nucleus, HMGB1 acts as a DNA chaperone and serves as a crucial factor in various cellular processes, including DNA replication and transcription, chromatin remodeling, V(D)J recombination, DNA repair, and the preservation of genomic stability (2, 3). In the cytoplasm, it binds to Beclin-1 protein to promote autophagy (4). When actively secreted by immune cells or passively released from necrotic cells into the extracellular space, HMGB1 functions as a DAMP. It triggers the innate immune response through its interaction with pattern-recognition receptors (PRRs), while simultaneously conveying danger signals to peripheral tissues via its conventional receptor pathways, RAGE (Receptor for End Products of Advanced Glycosylation) and TLR4 (5).

## 2 Overview of HMGB1

### 2.1 Biological properties of HMGB1

#### 2.1.1 Main structure of HMGB1

**Figure 1:**
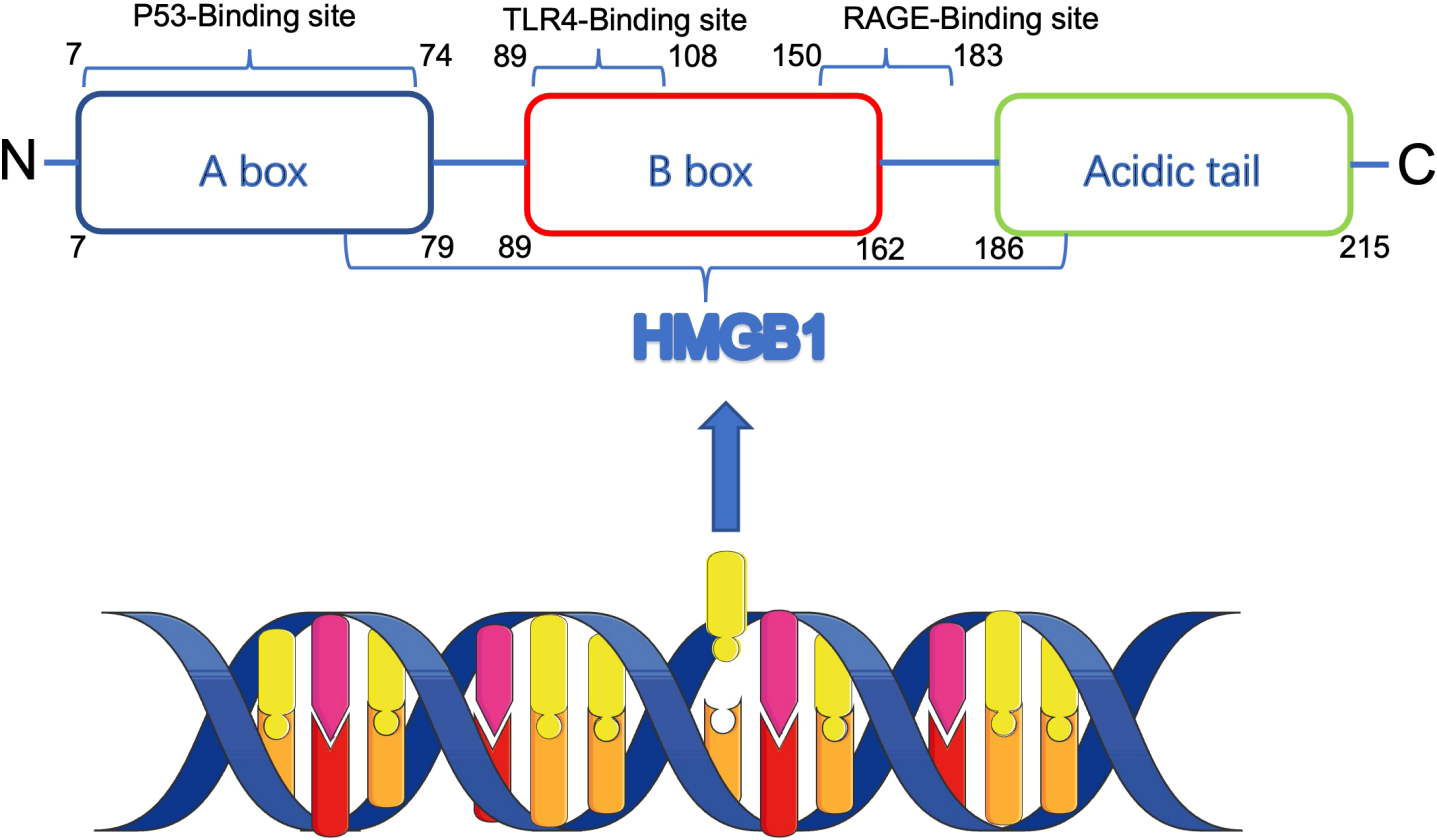
Schematic structure of HMGB1 protein and its receptor interactions. HMGB1 contains two DNA-binding domains (A-box and B-box) and an acidic C-terminal tail. The B-box domain mediates pro-inflammatory signaling via TLR4 and RAGE receptors, while the A-box and C-terminal tail possess anti-inflammatory properties. HMGB1 functions vary depending on redox status, localization, and post-translational modifications.

The HMGB1 gene, situated on chromosome 13 at position q12, comprises five exons interspersed with four introns. The HMGB1 protein is a highly conserved chromatin-associated protein with a molecular weight of approximately 25 kD, consisting of 215 amino acids. The protein consists of three major structural domains: two DNA-binding HMG-box structural domains (A-box: 7 - 79aa, B-box: 89 - 162aa) and an acidic C-terminal tail (186 - 215aa). In the B-box structural domain, there are Toll-like receptor 4 (TLR4) binding sites (89 - 108aa) and receptor for advanced glycosylation end products (RAGE) binding sites (150 - 183aa) (6, 7).The B-box interacts with TLR4 and RAGE through these binding sites, thereby inducing the release of inflammatory factors and promoting HMGB1 pro-inflammatory activity enhancement (8). In contrast, the A-box functions as a selective antagonist of the B-box (9), effectively suppressing its proinflammatory activity, while the C-terminal tail is able to bind the A-box and enhance its anti-inflammatory activity (10). The domain organization and receptor interactions of HMGB1 are schematically summarized in **Figure 1**. HMGB1 is highly conserved during evolution, but its specific function is influenced by factors such as chemical modification, subcellular localization, redox state, and receptor, and thus it exhibits a wide range of different biological functions. Some of these functions are similar to specific biological features proposed by Hanahan and Weinberg and are related to their proposed cancer marker signature (11).

#### 2.1.2 Mechanisms of HMGB1 release

In addition to its role in the nucleus, when HMGB1 is released outside the cell, it acts as a DAMP that regulates a variety of cellular life processes, such as cell differentiation, inflammatory response, and cell migration, through different receptors or direct uptake, etc. (12) HMGB1 can be released outside the cell by two main mechanisms: active secretion and passive release. When immune cells, fibroblasts or epithelial cells are exposed to external stimuli (e.g., LPS, CpG-DNA, TNF-α, NO, IFN-α, etc.), HMGB1 can be actively secreted through ROS, calcium ions, or NO signaling pathway; in addition, under the induction of cell death (e.g., necrosis, apoptosis, autophagy) or injury by chemotherapy, radiotherapy, hypoxia, etc., HMGB1 can be released through PARP1, RIP3 and cysteine. RIP3 and cysteoaspartase-dependent mechanisms.

#### 2.1.3 HMGB1 gene and regulation

The tissue harboring the HMGB1 gene, which has remained highly conserved throughout human evolution, comprises two HMG boxes (box A and box B) and an acidic C-terminal tail. Notably, box A and box B seem to be integrated with distinct genes, each encoding one of the boxes (13). Conversely, the human genome possesses functional HMGB1 genes (14), alongside several HMGB1 pseudogene sequences that lack introns (15).

In the human genome, the HMGB1 gene is regulated by a robust TATA-less promoter. Upstream of this promoter lies a silencer element capable of repressing HMGB1 activity, whereas the intronic regions of the HMGB1 gene harbor enhancers that can augment promoter activity (16). The transcriptional regulation of HMGB1 is further modulated by the P53 family, which acts to repress its activity, potentially through interactions with the CCAAT-binding transcription factor 2 (CTF2) (17). Additionally, HMGB1 facilitates the binding of P53 to linear DNA, thereby enhancing P53 activity during transcription (18). Within the nuclei of eukaryotic cells, HMGB1 is ubiquitously present as a DNA chaperone and participates in various critical DNA regulatory processes. It modulates gene expression by associating with histones and DNA, maintaining nucleosome stability, and promoting nucleosome sliding to expose DNA at transcriptional sites (19). In its role as a DNA chaperone, HMGB1 interacts with linear DNA, inducing a specific helical conformation that modulates the DNA’s sensitivity to various cytokines involved in processes such as DNA replication, transcription, repair, chromatin remodeling, and V(D)J recombination in both T and B cells (3, 19). HMGB1 enhances the looped configuration of DNA by increasing its flexibility, thereby facilitating the recruitment of additional transcription factors and promoting their interactions with transcription factors bound to distant regulatory sequences (20). Upon binding to DNA, HMGB1 undergoes various post-translational modifications, including phosphorylation, acetylation, and oxidation (21). As a DNA chaperone, HMGB1 is crucial in DNA repair and the response to DNA damage. Its deletion often results in increased DNA damage, heightening the cellular response to chemotherapy and radiotherapy. In the context of non-homologous end-joining (NHEJ) repair, HMGB1 is involved in the repair process mediated by DNA break ends associated with DNA-PKcs (22, 23).

### 2.2 Receptors and signaling pathways of HMGB1

**Figure 2:**
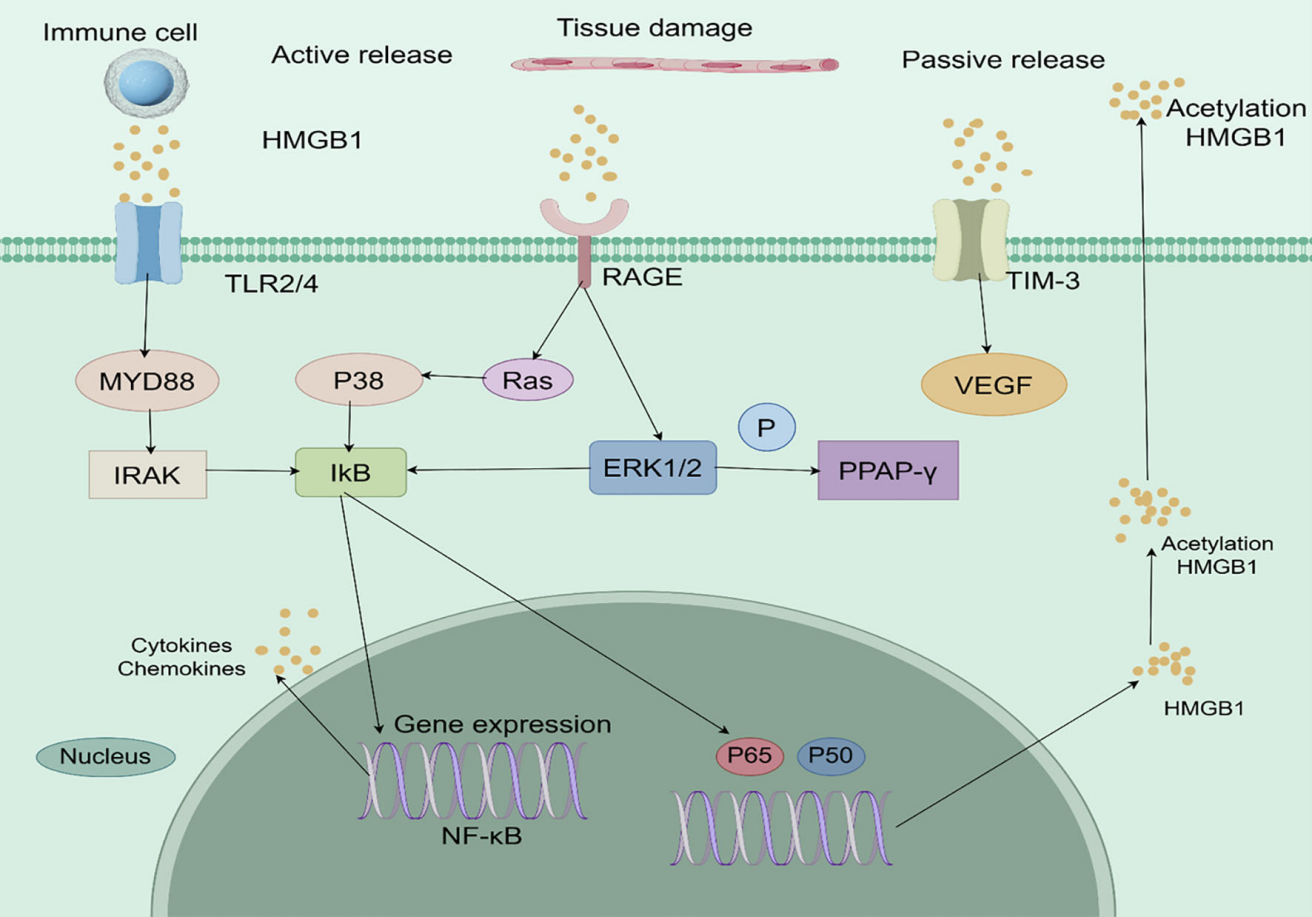
Analytical overview of HMGB1-mediated signaling pathways in the tumor microenvironment. HMGB1 is actively secreted by immune cells or passively released from damaged or necrotic cells. It binds to pattern recognition receptors such as TLR2/4 and RAGE, triggering downstream signaling cascades including MyD88-IRAK and Ras/ERK/PPAR-γ pathways. These interactions lead to NF-κB activation, pro-inflammatory cytokine production, and VEGF-mediated angiogenesis, collectively promoting tumor progression, immune evasion, and therapy resistance. .

When HMGB1 is released extracellularly, it binds to a variety of receptors to induce the DAMP response. The classical receptors for HMGB1 include Receptor for Advanced Glycation Endproducts (RAGE), TLRs (TLR2, TLR4, TLR9), CD24, CXCR4, and T-cell immunoglobulin mucin 3 (TIM - 3), among others, CD24, CXCR4, and T cell immunoglobulin mucin 3 (TIM - 3) (24). The complex signaling networks activated by extracellular HMGB1 in the tumor microenvironment are illustrated in **Figure 2**.

#### 2.2.1 RAGE

RAGE is an immunoglobulin with a molecular weight of approximately 45 kDa that is highly expressed during embryonic development, but its expression in a variety of tissues gradually declines in adulthood (25).RAGE is the first receptor shown to bind HMGB1 (26), and the HMGB1-RAGE axis plays different roles in cell death, cell adhesion, cell proliferation, and differentiation, and interacts with other HMGB1 receptors such as TLRs and NF-κB. Studies have shown that activation of RAGE has an important role in cancer development. By inhibiting p44, p42, and p38 and activating SAP and JNK MAP kinases, RAGE promotes tumor cell proliferation, invasion, and enhances matrix metalloproteinase expression (27). In addition, RAGE can be activated by HMGB1 released from necrotic tumor cells, which induces autophagy and inhibits apoptosis, thus enhancing cell resistance (28). In pancreatic cancer, RAGE can be activated by members of the S100 protein family to induce pancreatic cancer cell proliferation and invasion (29). Currently, The mechanistic intricacies underlying the HMGB1-RAGE signaling pathway in oncogenesis remain incompletely elucidated, but the mechanism by which HMGB1 directly activates RAGE and plays a promotional role in a variety of cancers could be a potential research direction for cancer therapy.

#### 2.2.2 TLRs

TLRs are pattern recognition receptors (PRRs) first identified in Drosophila and innate immunity relies heavily on these elements as its fundamental constituents (30).TIRS are classified into TLR2, TLR4, and TLR.HMGB1 can bind to TLR2 and participate in aseptic inflammatory responses (11). In addition, the interaction between HMGB1 and TLR2 plays a key role in the pathologic process of several diseases. For example, in rheumatoid arthritis, HMGB1 exacerbates the inflammatory response by upregulating the expression of TLR2 and IL - 23 in CD14+ monocytes and promoting the differentiation of Th17 cells (31). This mechanism highlights the pivotal role of the HMGB1-TLR2 axis in controlling immune responses and inflammatory processes. Thus, HMGB1 participates in and regulates aseptic inflammatory responses by binding to TLR2, a mechanism that has been demonstrated in a variety of inflammatory diseases and provides a potential target for the treatment of related disorders. TLRs recognize a variety of danger signals, These molecular signals, including pathogen-associated molecular patterns (PAMPs) and damage-associated molecular patterns (DAMPs), trigger the body’s innate immune defenses to combat potential threats (32). TLRs primarily exert their functions via two distinct signaling pathways: the MyD88-dependent and MyD88-independent routes. The former is crucial for the generation of inflammatory factors, whereas the latter significantly contributes to the maturation processes of type I interferon modulators and dendritic cells.HMGB1 can act as a ligand for TLRs, and upon binding to it, it activates the NF-κB signaling pathway to induce the production of cytokines and chemokines, and the production of cytokines and chemokines. and chemokine production, thereby triggering inflammatory and immune responses (33). The release of HMGB1 from necrotic keratinocytes triggers a TLR4-dependent inflammatory response and promotes the development of skin cancer (34). In cases of localized breast cancer metastasis, the levels of TLR4 and MyD88 signaling components were markedly elevated compared to both normal tissue and benign breast tumor samples (35).which may be associated with the activation of the TLR4-mediated paracrine signaling pathway, which in turn promotes tumor progression. In melanoma, increased expression of the TLR4 agonist HMGB1 enhances the proliferation of tumor cells, but its correlation with patient survival and melanoma progression is low. HMGB1 binds to TLR9 and enhances its recognition of CpG-DNA, which promotes immune responses (36). This binding not only helps to recognize exogenous pathogen DNA, but may also have an impact on autoimmune diseases. In autoimmune diseases such as systemic lupus erythematosus (SLE), the level of HMGB1 is significantly elevated and is closely associated with the pathological process of the disease. It has been found that HMGB1 This mechanism potentiates TLR9-mediated immune activation through facilitating DNA accumulation within endosomes, consequently intensifying the inflammatory cascade (37, 38). This mechanism suggests that HMGB1 may be a potential target in the treatment of diseases such as SLE. In addition, the interaction of HMGB1 with TLR9 may also affect the function of other immune cells. For example, it has been shown that HMGB1 is able to regulate dendritic cell maturation and antigen-presenting functions through binding to TLR9, thereby affecting adaptive immune responses (25). This mechanism is also important in cancer immunotherapy as it may enhance therapeutic efficacy by augmenting anti-tumor immune responses. Thus, HMGB1 significantly enhances the recognition and response of the immune system to CpG-DNA by binding to TLR9. This mechanism serves a vital function in both infectious and autoimmune diseases, while simultaneously presenting fresh perspectives and potential treatment avenues for malignancies and other conditions through immunotherapy.

#### 2.2.3 TIM3

TIM3, a member of the T cell immunoglobulin and mucin domain (TIM) family, was initially identified on the surface of interferon gamma (IFNγ)-producing T helper type 1 (Th1) cells and is involved in macrophage activation and the regulation of autoimmune diseases (39). Follow-up studies have revealed that TIM - 3 is also present on the surface of Th17 cells, Macrophages and dendritic cells are crucial for modulating innate immune responses and maintaining inflammatory homeostasis within the organism (40, 41). In addition, TIM - 3 is active in innate immunity and in a variety of cancers. In hepatocellular carcinoma, TIM - 3 induces the activation of tumor-associated macrophages (TAMs) in the tumor microenvironment and promotes the conversion of M1-type macrophages to M2-type macrophages, which in turn promotes cancer cell growth (42). In tumor immunotherapy, inhibition of programmed cell death factor 1 (PD - 1) and its ligand PD-L1 has shown significant clinical efficacy. Studies have shown that blockade of The PD - 1/PD-L1 signaling axis plays a crucial role in reactivating T-cell-mediated immunity, thereby potentiating the host’s defense mechanisms against malignant cells. Dual blockade of TIM - 3 and PD - 1 significantly enhances anti-tumor immune responses. Inhibition of TIM - 3 expression enhances the anti-tumor effects of programmed cell death factor 1 (PD - 1) (43). HMGB1 interacts with TIM - 3, triggering the release of vascular endothelial growth factor (VEGF). This process facilitates tumor angiogenesis, thereby enhancing the sustenance and expansion of acute myeloid leukemia (AML) cells (11). In addition, anti-TIM-3 monoclonal antibody treatment improved the efficacy of chemotherapy in hormonal mice (44). The above studies suggest that antitumor therapy combining HMGB1 and TIM - 3 has become a potential research direction for tumor immunotherapy.

## 3 Role of HMGB1 in cancer

**Figure 3:**
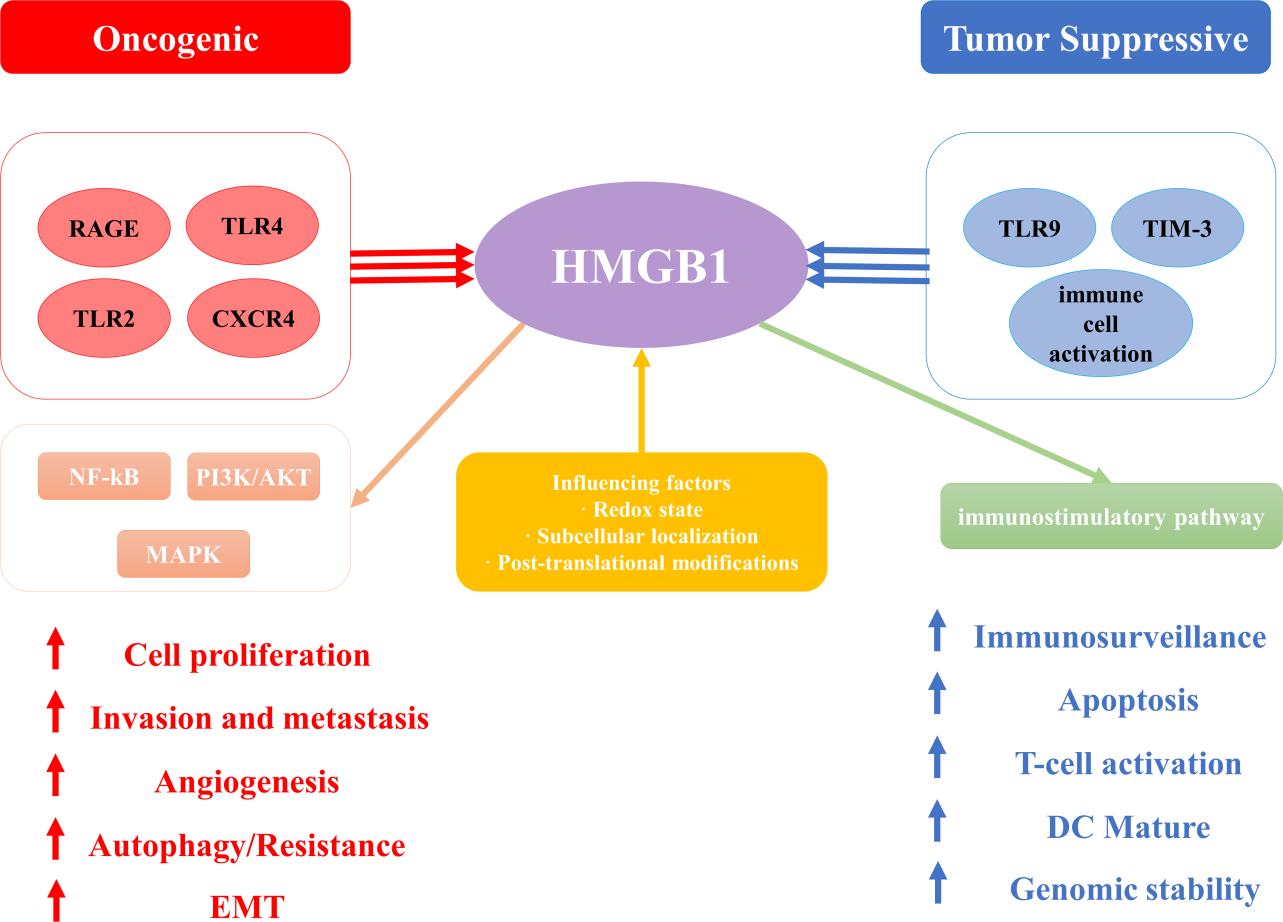
The schematic illustration elucidates the paradoxical dual role of High Mobility Group Box 1 (HMGB1) in cancer pathogenesis, emphasizing its context-dependent oncogenic and tumor-suppressive functions. .

The functional duality of HMGB1 is consistent with Hanahan’s cancer hallmarks (45), demonstrating context-dependent oncogenic or tumor-suppressive effects. As illustrated in **Figure 3**, the signaling outcomes of HMGB1 are determined by receptor interactions (such as RAGE/TLRs promoting tumorigenesis versus TIM - 3/TLR9 facilitating immune activation), redox state, and subcellular localization. These mechanisms are fundamental to HMGB1’s involvement in proliferation, metastasis, therapy resistance, and immune modulation in various malignancies.

With further research on HMGB1, HMGB1 has been shown to be associated with cancer hallmark features proposed by Hanahan (45). These hallmark features include ten abilities such as maintaining proliferative signaling, evading growth inhibition, resisting cell death, acquiring unlimited proliferative capacity, inducing angiogenesis, promoting tumor inflammation, enhancing invasion and metastasis, altering cellular metabolism, evading immune surveillance, and affecting genetic stability (11). Previous research has established that HMGB1 plays a pivotal role in the initiation process, advancement, and therapeutic management of numerous cancer types, including colorectal cancer (46), pancreatic cancer (47), non-small cell lung carcinoma (NSCLC) (48), melanoma (49), ovarian cancer (50), breast cancer (51), and hepatocellular carcinoma (52). However, HMGB1 does not always promote cancer progression, and in some cases it appears to exhibit conflicting effects. In some cases, HMGB1 does promote cancer progression and invasion. For example, in desmoplasia-resistant prostate cancer, HMGB1 promotes tumor progression by binding to TNFR1 and activating the NF-κB signaling pathway (31), and furthermore, high expression of HMGB1 in rectal cancer is closely associated with cancer progression and metastasis, and its down-regulation significantly inhibits the proliferation of rectal cancer cells and induces their apoptosis (53). However, it may also inhibit tumor progression through different mechanisms. For example, its function is influenced by the redox state: reduced HMGB1 acts as a chemokine, whereas HMGB1 containing disulfide-bonded structures possesses pro-inflammatory cytokine properties (54). This redox state switch may play an inhibitory role in tumor progression in some cases. Depending on its subcellular localization and modification status, HMGB1 may act as either a pro- or an oncogenic factor (55).

### 3.1 Role of HMGB1 in tumorigenesis and progression

HMGB1 plays an important role in the development and progression of many cancers. HMGB1, a non-histone nuclear protein, can be translocated to the extracellular space through passive release mechanisms under conditions of cellular stress, injury, or death. The release of HMGB1 has been suggested to be a “danger signal” that can affect The capacity of malignant cells to evade the body’s immune defenses (56). The release of HMGB1 is considered a “danger signal” that can affect the ability of cancer cells to evade host immune surveillance (56). On the one hand, HMGB1 exerts pro-oncogenic effects by enhancing anti-apoptotic mechanisms, inflammatory responses, immune escape and aerobic glycolysis (57, 58). Meanwhile, it also participates in tumorigenesis and progression through TLR4- and RAGE-mediated inflammatory responses (34, 59), enhances the expansion of diverse immune cell populations within the tumor microenvironment (TME), and activates relevant cytokine signaling pathways, thereby promoting tumor transformation, supporting tumor growth, and facilitating tumor invasion and metastasis. Under the stimulation of TME-induced hypoxia, inflammation, and injury, intracellular HMGB1 is further released to accelerate the inflammatory response and increase the release of RAGE in TME, The activation of the HMGB1/RAGE/NF-κB signaling cascade triggers the upregulation and secretion of vascular endothelial growth factor along with its receptor, consequently facilitating tumor angiogenesis and metastatic progression (60).Taguchi and David (11)found that the proliferative and metastatic abilities and matrix metalloproteinase activity were decreased to different degrees by inhibiting the expression of HMGB1/RAGE in tumor tissues.

In acute myeloid leukemia, released HMGB1 promotes the proliferation of AML cells by influencing TNF-α production and interleukin 1β (IL - 1β) secretion, which promotes the release of stem cell factors (11). Hypoxia is one of the hallmarks of a variety of solid tumors, and it promotes tumor invasion into surrounding tissues and even distant metastasis by inducing the hypoxia-inducible factor (HIF) family of transcription factors, This pathway activates the expression of multiple growth factors, including vascular endothelial growth factor (VEGF), in addition to angiopoietin 2 (Ang-2) and fibroblast growth factor (FGF) (60). However, the exact mechanism of hypoxia’s effect on cancer is unclear.

Previous studies have shown that under hypoxic conditions in hepatocellular carcinoma, HMGB1 can be passively released and induce caspase-1 activation through activation of TLR4 and RAGE signaling pathways, thus promoting cancer cell invasion and metastasis (61). In melanoma, hypoxia leads to cell necrosis and release of HMGB1, which interacts with RAGE to activate M2-type macrophages to secrete interleukin-10 (IL - 10), thereby promoting melanoma growth and metastasis (49). In non-small cell lung cancer (NSCLC), HMGB1 activates the PI3K/Akt and NF-κB signaling pathways and induces matrix metalloproteinase-9 (MMP - 9) expression, which enhances the invasiveness and migratory ability of NSCLC cells (48).

In summary, HMGB1 activates multiple inflammatory factors in TME by activating signaling pathways such as RAGE, TLR4, and PI3K, thereby promoting tumor growth, facilitating cellular invasion and metastatic spread, while concurrently contributing to therapeutic resistance in cancer treatment. However, the interrelationships between HMGB1 and its different ligands remain unclear. Therefore, A more profound comprehension of HMGB1’s function within the TME can be achieved and tumor immunity will help us better develop novel tumor immunotherapy strategies targeting HMGB1.

### 3.2 Anti-tumor effects of HMGB1

On the other hand, the anti-tumor properties of HMGB1 are primarily manifested through its capacity to augment the immune system’s recognition and targeting efficiency against malignant cells. The compound stimulates the immune system, particularly dendritic cells (DCs) and T lymphocytes, by interacting with Toll-like receptor (TLR) and receptor for advanced glycosylation end-products (RAGE), thereby boosting the organism’s immune defense against tumor growth (62).The role of HMGB1 in radiation therapy has been widely noted, and radiation therapy not only directly induces cell death, but also by releasing HMGB1 and other “danger signals” to induce anti-tumor responses of the immune system (63).The release of HMGB1 helps immune cells to recognize tumor cells, which enhances the therapeutic effect of radiotherapy. In addition, HMGB1 can exert anti-tumor effects by inducing apoptosis and inhibiting proliferation of tumor cells. Some studies have shown that HMGB1 can inhibit tumor cell growth by interacting with TLR4 and triggering the production of immunosuppressive proteins (56). Finally, HMGB1 can also synergize with chemotherapeutic agents to exert antitumor effects in some cases.HMGB1 can enhance antitumor immune responses by promoting the release of interferon and other cytokines when combined with paclitaxel (64).

Also, HMGB1 can activate or enhance anti-tumor immunity in a variety of ways during cancer development and treatment. Within the MCF - 7 breast carcinoma cell line, HMGB1, which possesses the LXCXE sequence, enhances the transcriptional capacity of cell cycle protein A inhibition by interacting with the Rb gene, inducing cell arrest and death in the G1 phase (65). When leukemia cells are stimulated by cytotoxicity produced by chemotherapeutic drugs, they release HMGB1 from the cells to resist the damage. And after the addition of HMGB1-neutralizing antibody treatment, leukemia cells have increased sensitivity to chemotherapy (66). In non-small cell lung cancer, doxorubicin can enhance tumor immunity and improve the effect of chemotherapy by enhancing the secretion of HMGB1 and CXCL11 (67). Decreased genetic stability is regarded as a major driver during cancer development and progression.HMGB1, as a DNA chaperone, plays an important role in the regulation of genetic stability. Deficiency of HMGB1 leads to decreased genomic stability, nucleosome release, and telomere shortening, which induces inflammatory responses and triggers innate immunity (68, 69). In addition, damage repair mediated by HMGB1 helps to restore genomic stability (70). Therefore, how to maintain the immune-stimulating, damage-repairing function of HMGB1 in cancer development is an important current challenge.

In summary, HMGB1 exerts dual anti-tumor effects by both directly suppressing tumor growth through cytotoxic mechanisms and enhancing immune-mediated anti-tumor responses. Given its multifaceted role, HMGB1 has emerged as a promising therapeutic target, offering new directions for the development of novel cancer treatment strategies. To further illustrate the context-dependent nature of HMGB1’s function, we have summarized its oncogenic and tumor-suppressive roles across various cancer types with representative examples (**Table 1**). This overview underscores the complexity of HMGB1-mediated signaling within the tumor microenvironment and highlights the need for tailored therapeutic approaches based on its distinct functional states.

**Table 1:** Oncogenic and tumor suppressor properties of HMGB1 with examples.

Property	Cancer Type	Mechanism/Pathway	Effect/Example	References
Oncogenic	Prostate cancer	HMGB1/RAGE, NF-κB, PI3K/AKT	Promotes EMT, proliferation, metastasis; contributes to ADT resistance	(71, 72)
Oncogenic	Bladder cancer	Cyclin A upregulation, autophagy induction	Facilitates cell cycle progression, proliferation, Chemoresistance	(73)
Oncogenic	Renal cell carcinoma	HMGB1/RAGE, VEGF induction	Enhances angiogenesis, autophagy-mediated drug resistance, invasion	(74)
Oncogenic	NSCLC	PI3K/AKT, NF-κB	Increases MMP - 9, invasiveness, and migration	(48)
Oncogenic	Melanoma	HMGB1-RAGE under hypoxia	Activates M2 macrophages, promotes metastasis	(49)
Tumor Suppressor	Breast cancer (MCF - 7)	Rb interaction	Induces cell cycle arrest and apoptosis	(65)
Tumor Suppressor	Multiple cancers (with chemotherapy)	Synergistic with paclitaxel, doxorubicin	Enhances antitumor immune response, chemotherapeutic efficacy	(64, 67)
Tumor Suppressor	General (DNA repair)	DNA chaperone activity	Maintains genomic stability, prevents mutation accumulation	(70)

### 3.3 Correlation between HMGB1 and resistance to chemotherapy and radiotherapy

HMGB1is a pivotal DAMP molecule, plays a critical role in mediating resistance to chemotherapy and radiotherapy across various tumor types. Increasing evidence indicates that HMGB1 contributes to therapeutic resistance through multiple molecular mechanisms, with its functional impact exhibiting notable tumor-type specificity.

In the context of chemotherapy resistance, HMGB1 predominantly exerts its effects by promoting autophagy. In bladder and prostate cancers, treatment with chemotherapeutic agents such as gemcitabine or paclitaxel induces the release of HMGB1, which subsequently enhances autophagic activity via direct interaction with Beclin-1. This activation of autophagy mitigates drug-induced cytotoxicity and facilitates tumor cell survival (75, 76). Moreover, HMGB1 can bind to RAGE and TLR4 receptors, activating downstream signaling pathways such as NF-κB and PI3K/Akt. This cascade promotes epithelial-mesenchymal transition (EMT), enhances tumor invasiveness, and further contributes to drug resistance (71, 77). In gastric cancer, elevated HMGB1 expression is strongly associated with resistance to cisplatin, while pharmacological inhibition of HMGB1 signaling markedly enhances cisplatin efficacy (78).

Regarding radiotherapy resistance, HMGB1 exhibits a dual and context-dependent role. On one hand, as a DAMP molecule, extracellular HMGB1 can activate dendritic cells and T lymphocytes, thereby stimulating antitumor immune responses (79). On the other hand, HMGB1 may attenuate radiotherapy sensitivity by promoting DNA damage repair and facilitating tumor cell recovery. For instance, in nasopharyngeal carcinoma, HMGB1 enhances the non-homologous end-joining (NHEJ) DNA repair pathway through interaction with Ku70, contributing to resistance to both radiotherapy and cisplatin. Suppression of HMGB1 expression has been shown to reverse this resistance phenotype (80). Similarly, in non-small cell lung cancer, downregulation of HMGB1 sensitizes tumor cells to radiation by inhibiting the TLR4/NF-κB signaling axis (81). Additionally, HMGB1 promotes the formation of a tumor-supportive microenvironment by activating survival-related signaling pathways and recruiting tumor-associated macrophages, further compromising the effectiveness of radiotherapy.

In conclusion, HMGB1 serves as a key regulatory hub in the development of resistance to chemotherapy and radiotherapy. Through the modulation of autophagy, enhancement of DNA repair, immune evasion, and remodeling of the tumor microenvironment, HMGB1 profoundly influences treatment outcomes. A deeper understanding of HMGB1-mediated resistance mechanisms may offer new avenues for overcoming therapeutic resistance and improving clinical prognosis in cancer patients.

## 4 Role of HMGB1 in urologic malignancies

HMGB1 plays a key role in the occurrence, development, and treatment resistance of malignant tumors of the urinary system (including prostate cancer, bladder cancer, and renal cell carcinoma). To systematically summarize its core mechanisms of action, we have summarized the signaling pathway interactions, molecular mechanisms, and clinical outcomes of HMGB1 in different urinary tumors (**Table 2**).

**Table 2:** The main signaling pathways and clinical implications of HMGB1 in malignant tumors of the urinary system.

Tumor	Interactors	Mechanism of action	Outcome	References
Prostate cancer	AR RAGE PI3K/AKT	Activate AR activity by promoting the nuclear translocation process of AR promotes prostate cancer metastasis in a paracrine form via HMGB1/RAGE regulating cell cycle proteins mediating epithelial mesenchymal transition	Progression Progression Metastasis	(82) (83) (71)
Renal cell carcinoma	RAGE Dendritic cells	Inducing the expression of vascular endothelial growth factor inhibiting the role of immunotherapy	Proliferation and Invasion Progression	(11, 74) (84)
Bladder cancer	Cell cycle protein A	Accelerates the transition of the uroepithelial cancer cell cycle from G to S1 phase	Progression	(73)

### 4.1 HMGB1 and prostate cancer

Prostate cancer is the second most common cancer worldwide and the fifth leading cause of cancer deaths in men, with approximately 1.4 million new cases and 370,000 deaths annually (85). Androgen deprivation therapy (ADT) represents the prevailing systemic therapeutic approach currently employed in clinical practice. However, after 18 – 36 months of treatment, the androgen receptor (AR) signaling pathway is reactivated in the majority of patients, inevitably progressing to castration-resistant prostate cancer (CRPC), which makes prostate cancer much more aggressive and lethal (86). The key step in this progression is the reactivation of the androgen receptor signaling pathway with a progressive rise in serum prostate-specific antigen. HMGB1, whose expression is significantly increased in prostate cancer specimens, has been reported to reactivate and interact with the AR signaling pathway to promote CRPC development in a non-androgen-dependent manner (82). Meanwhile, androgen deprivation therapy (ADT) induces HMGB1 secretion and promotes prostate cancer metastasis in a paracrine form via HMGB1/RAGE (83). In further studies, it was found that HMGB1 induces epithelial mesenchymal transition mainly by promoting overexpression of various matrix metalloproteinases with RAGE/NF-κB, which leads to prostate cancer metastasis (72). It can also activate the PI3K/AKT signaling pathway, which promotes prostate cancer growth and metastasis by regulating cell cycle proteins mediating epithelial mesenchymal transition (71). The addition of glycyrrhizin can inhibit this epithelial mesenchymal transition, suggesting that glycyrrhizin may be useful for the treatment of metastatic prostate cancer (87). In contrast, verbascoside inhibits HMGB1 expression and downregulates TGF-β-related epithelial mesenchymal transition to reduce prostate cancer proliferation and invasion (88). Recent studies have found a correlation between HMGB1 and AR receptor activation. gankyrin can cause prostate cancer progression by upregulating octamer-binding protein (NONO) expression, reactivating AR receptors, inducing HMGB1 transcription, and then promoting tumor-associated macrophage aggregation (89). HMGB1 significantly contributes to the advancement of prostate cancer while also is a huge hindrance during prostate cancer treatment. In androgen-independent prostate cancer, HMGB1 is released after gemcitabine treatment and promotes autophagy in interaction with Beclin-1, leading to androgen-independent prostate cancer resistance (75). Interestingly, the same large amount of HMGB1 was released in dead cells after treatment with doxorubicin, which mediated the survival of chemoresistant tumor cells through TLR4/RAGE-sCLU (90). And a variety of chemotherapeutic agents can lead to cell death releasing HMGB1 and inducing chemotherapeutic drug resistance in this way. Thus, we can hypothesize that in any of the cancers that have developed resistance, HMGB1 may synergize with each other to promote chemotherapeutic drug resistance in these two ways described above. When we administered glycyrrhizin or other HMGB1-neutralizing antibodies to desmoplasma-resistant prostate cancer cells that had developed resistance to paclitaxel, and then treated the cells again with paclitaxel, their sensitivity to paclitaxel was significantly restored (76). As mentioned earlier, ADT induces HMGB1 secretion, which promotes prostate cancer progression. Whereas, during treatment, enzalutamide induced elevated levels of HMGB1 expression, recruitment and activation of tumor-associated macrophages to secrete interleukin 6 led to enzalutamide resistance (91). In a further study, The single nucleotide polymorphisms of HMGB1 were identified to be correlated with adverse clinical outcomes in prostate cancer, including higher D’Amico classification, higher pathologic Gleason subgroups, and higher pathologic stage (92). In a multivariate study that included 168 patients who underwent radical prostatectomy for prostate cancer, HMGB1 expression was associated with pathological stage of prostate cancer, Gleason score, and pathological stage, and could also be an independent prognostic factor for biochemical recurrence-free survival after radical prostatectomy (93). In summary, HMGB1 exerts diverse functions in the advancement, metastatic potential, and therapeutic recalcitrance of prostate cancer, positioning it as a promising candidate for both targeted therapy and prognostic evaluation. Future studies could further explore the specific mechanisms of HMGB1 in prostate cancer to develop new therapeutic strategies. In summary, HMGB1 plays a multifaceted role in prostate cancer progression, invasion and treatment resistance, becoming a potential therapeutic target and prognostic marker. Future studies can further explore the specific mechanisms of HMGB1 in prostate cancer to develop new therapeutic strategies.

### 4.2 HMGB1 and bladder cancer

HMGB1 exhibits tumor-promoting effects in a wide range of cancers, and its specific mechanisms are gradually being revealed in bladder cancer. Bladder cancer is one of the top ten most common cancers worldwide and places a heavy burden on patients and global healthcare systems due to its highly heterogeneous nature and high recurrence rate (94). Therefore, there is a need to introduce new anticancer strategies with a view to positively impacting the prognosis of patients. In an earlier study, YANG et al. noted that the expression level of HMGB1 was significantly correlated with the overall survival of bladder cancer patients and showed significant differences in the stage and grading of bladder cancer (95). In addition, HMGB1 accelerates the transition of the uroepithelial cancer cell cycle from G to S1 phase by promoting the expression of cell cycle protein A, which promotes the growth of bladder cancer (73). Research indicates that HMGB1 modulates key cellular mechanisms, including programmed cell death and proliferation, thereby influencing the proliferative capacity and metastatic potential of bladder cancer cells (96). In addition, the expression level of HMGB1 is closely related to the pathological grading and prognosis of bladder cancer, which makes it a potential diagnostic and prognostic biomarker (97).

Usually, after surgery, we use bladder perfusion therapy with drugs such as gemcitabine, mitomycin, and epirubicin to reduce the recurrence rate of bladder cancer. However, as previously mentioned multiple chemotherapeutic agents can lead to the release of HMGB1, which leads to the development of drug resistance, and this is no different in bladder cancer.HMGB1 is passively released in response to gemcitabine, which attenuates the anticancer effect of gemcitabine by mediating an increase in autophagy (73). For invasive bladder cancer, we currently usually use radical cystectomy, but this procedure significantly reduces the quality of life of patients. Therefore, some patients choose a triple or multiple combination treatment regimen of surgery, chemotherapy, and radiotherapy (98).Shrivastava et al. suggested that lowering the expression of HMGB1 in bladder cancer could effectively inhibit autophagy and histiocyte repair, and enhance the efficacy of radiotherapy (99). Therefore, it is reasonable to infer that for patients who cannot undergo or cannot tolerate radical cystectomy, the choice of triple or multiple combination therapy along with inhibition of HMGB1 expression may be helpful for patient survival. In addition, when facing patients with suspected bladder cancer or postoperative bladder cancer, we often use cystoscopy coupled with biopsy for screening and diagnosis, but this is an invasive test and not all hospitals can carry out the comparison of urinary HMGB1 expression levels of patients with bladder cancer, patients with urinary tract infections, and healthy people, the expression of urinary HMGB1 in patients with bladder cancer is significantly higher than that of the two, and high-level tumors were also significantly different from lower grades (100). Thus, HMGB1 serves as an effective early indicator for predicting the prognosis of patients with bladder cancer, and at the same time, inhibition of HMGB1 may enable bladder cancer patients to obtain greater benefits from systemic therapy.

### 4.3 HMGB1 and renal cell carcinoma

Renal cell carcinoma is the most common renal malignancy, with a complex biology and high susceptibility to recurrence and metastasis (101). Because renal cell carcinoma itself exhibits strong drug resistance, radiotherapy, which is currently used routinely, has little effect on patient survival. Therefore, an in-depth understanding of the mechanisms of renal cell carcinoma occurrence, progression, and treatment has led to the proposal that targeting HMGB1 has significant benefits for patients with renal cell carcinoma. In renal cell carcinoma, overexpression of HMGB1 is always closely associated with the malignancy of the tumor (102). In a clinicopathological analysis including 80 patients with renal cell carcinoma, elevated HMGB1 levels were significantly associated with unfavorable clinical outcomes, and this manifestation appeared to be achieved by affecting the receptor product of glycosylation endpoints(RAGE) (74). HMGB1/RAGE promotes renal cell carcinoma proliferation and invasion by inducing the expression of vascular endothelial growth factor (VEGF), and at the same time promotes renal cell carcinoma cell autophagy to enhance drug resistance (11). Therefore, we can inhibit angiogenesis and metastasis of renal cell carcinoma by targeting HMGB1 and use it in combination with other renal cell carcinoma therapies to prolong patient survival. Immunotherapy, the latest cancer treatment, which treats cancer by stimulating the recovery of the body’s autoimmune function, has not yielded satisfactory results in renal cell carcinoma patients. In the study of immune evasion in renal cell carcinoma, it was found that HMGB1 did not directly affect adaptive immunity, but promoted the proliferation of myeloid-derived suppressor cells (MDSCs) by interfering with the differentiation of dendritic cells, inhibiting the role of immunotherapy in renal cell carcinoma (84). In addition, Zhong et al. found that low expression of miR-505 led to overexpression of HMGB1, which promoted the proliferation of renal cell carcinoma (103). Research findings indicate that HMGB1 may serve as both a diagnostic marker and treatment focus for renal cell carcinoma. Furthermore, based on these findings, targeting HMGB1 and combining it with immunotherapy may be a promising therapeutic strategy when facing patients with advanced renal cell carcinoma.

## 5 Conclusion and outlook

HMGB1, a key DAMP, significantly influences the onset, progression, and treatment resistance of urological cancers. It fosters chemoresistance and radioresistance by promoting autophagy, enhancing DNA repair, activating survival pathways, and altering the tumor immune microenvironment. In prostate and bladder cancers, HMGB1 released during chemotherapy boosts autophagy via Beclin-1, reducing drug toxicity and aiding tumor survival. It also engages RAGE and TLR4 receptors to activate NF-κB and PI3K/Akt pathways, driving EMT and resistance. In radiotherapy, HMGB1 both activates anti-tumor immunity and aids DNA repair, interacting with Ku70 in nasopharyngeal carcinoma and modulating the TLR4/NF-κB axis in lung cancer. These findings highlight the central role of HMGB1 in orchestrating tumor cell survival in the face of cytotoxic therapies.

Despite these advancements, several critical challenges persist. Firstly, the tumor-type specificity of HMGB1-mediated resistance mechanisms remains inadequately elucidated, particularly within the diverse immunological and metabolic microenvironments. Secondly, the dualistic nature of HMGB1, functioning both as a tumor promoter and an immune activator, highlights the complexity of its role across various stages of tumor progression and therapeutic intervention. Thirdly, although preclinical studies suggest the potential of targeting HMGB1 or its downstream pathways to enhance therapeutic efficacy, there is a notable absence of clinically approved HMGB1 inhibitors, and the safety and specificity of these approaches necessitate further validation.

In conclusion, HMGB1 functions as a pivotal nexus connecting inflammation, immune modulation, and treatment resistance in urologic and other malignancies. A more thorough understanding of its signaling pathways and cellular interactions could reveal novel therapeutic opportunities. Future research should aim to elucidate the context-specific roles of HMGB1 within the tumor microenvironment and to develop safe and effective HMGB1-targeted agents. These agents could potentially be used in conjunction with existing chemotherapy, radiotherapy, or immunotherapy strategies to enhance patient outcomes.
