# Correction: Mechanism of action of HMGB1 in urologic malignancies

**DOI:** 10.3389/fonc.2025.1735134

**Published:** 2025-12-02

**Authors:** Dandan Li, Xu Lei, Lanlan Zhao, Qingqing Fu, Yao Chen, Xiaorong Yang

**Affiliations:** 1 Department of Pathology, Affiliated Hospital of Zunyi Medical University, Zunyi, Guizhou, China; 2 School of Basic Medical Sciences, Zunyi Medical University, Zunyi, Guizhou, China; 3 Department of Urology, Third Affiliated Hospital of Zunyi Medical University, Zunyi, China

**Keywords:** high mobility group protein 1 (HMGB1), prostate cancer, renal cell carcinoma, urologic tumors, bladder cancer

An incorrect **Funding** statement was provided. The correct funder is “Guizhou Science and Technology Foundation (Qiankehe basic-ZK[2023] General 566, Qiankehe basic-ZK[2021] General 383) and Scientific Research Project of Guizhou Provincial Health Commission (gzwkj2024-206, gzwkj2023-034).”

The original version of this article has been updated.

